# Investigation and analysis of four countries’ recalls of osteosynthesis implants and joint replacement implants from 2011 to 2021

**DOI:** 10.1186/s13018-022-03332-w

**Published:** 2022-10-07

**Authors:** Yang Wang, Kai Xu, Yuchen Wang, Weijie Ye, Xinyi Hao, Shouli Wang, Kun Li, Jun Du

**Affiliations:** 1 Department of Orthopedic Surgery, The First Affiliated Hospital of Soochow University, No. 899 Ping Hai Road, Suzhou, 215000 China; 2 Department of Pathology, School of Biology and Basic Medical Sciences, Soochow University, Suzhou, 215123 China; 3 Suzhou Medical Device Safety Evaluation Center, Suzhou, 215123 China; 4 Biopharmagen Corp., Fangzhou Suzhou, No. 88 Dongchang Road, Suzhou, 215127 China; 5 Department of Orthopedic Magnetic Resonance Chamber, The First Affiliated Hospital of Soochow University, No. 899 Ping Hai Road, Suzhou, 215000 China

**Keywords:** Medical device recalls, Osteosynthesis implants, Joint replacement implants, Safety evaluation, McKinsey 7S model

## Abstract

**Background:**

Medical devices are used in almost all orthopedic surgical subspecialties, and the frequency of adverse events is increasing, which should not be ignored. To provide suggestions on how to avoid implant recalls from the perspective of manufacturers, medical institutions and supervisions, as well as how to respond promptly to adverse events.

**Methods:**

The research extracted recalls of osteosynthesis implants and joint replacement implants from January 1, 2011, to June 30, 2021, in the CNMPA, FDA, HC and ATGA websites and collected the information on device name, recall time, recall class, recall manufacturer, device classification and affected areas. Moreover, the McKinsey 7S model and fishbone diagram were used to analyze recall reasons.

**Results:**

A total of 315 cases of osteosynthesis implants and 286 cases of joint replacement implants were reported in China, the USA, Canada and Australia. The recalls number from 2016 to 2021 was more than that from 2011 to 2015 for osteosynthesis implant (*p* = 0.012) and joint replacement implant (*p* = 0.002), and both mainly focused on class II (76.19% and 78.32%). There were statistical differences in the four countries for both implants (*p* = 0.000), especially osteosynthesis implant between China and the USA (*p* = 0.000), China and Canada (*p* = 0.001), the USA and Australia (*p* = 0.002), and joint replacement implant between China and Australia (*p* = 0.000).

**Conclusions:**

To avoid the recalls of such implants, manufacturers should strictly select implant materials and components, develop detailed labels and instructions, severely control the packaging process and establish the integrity of medical device data. Medical institutions should standardize procurement procedures, use qualified equipment and train medical workers. It also requires supervisions to conduct premarket safety assessments. In addition, regulators should strengthen supervision and establish reporting systems to deal with the occurrence of adverse events promptly.

**Supplementary Information:**

The online version contains supplementary material available at 10.1186/s13018-022-03332-w.

## Background

Medical devices are used in almost all orthopedic surgical subspecialties. Estimations for the global medical device market have increased from $260 billion in 2006 to more than $380 billion in 2016 [1]. Although many clinical problems have been addressed by medical devices, the frequency of adverse medical device events is increasing, which should not be ignored [2, 3].

Bone tissue has a remarkable ability to regenerate and heal by itself [4]. However, large bone defects and complex fractures remain one of the major challenges the medical community facing, with the current treatment focusing on the metal implants for structural and mechanical support [4]. Osteosynthesis implant is a non-active surgical implant that supports bone, cartilage, tendons and ligaments, promotes bone healing or ensures the stability of osteotomy and is arthroplasty to protect the bone neck and is often used to treat fractures and bone tumors [5]. Another joint replacement implant, also known as an artificial joint prosthesis, includes ancillary implanted components and materials, connected to the corresponding human bones and replacing the diseased joints, thus providing functions similar to natural joint [6].

In 1980, Thomas J. Peters and Robert H. Waterman developed a new management model to better manage the enterprise—McKinsey 7S model, which consists of Strategy, Structure, System, Staff, Skills, Style and Share Values [7]. After continuous development, the McKinsey model has been applied in medical fields, such as the management of opioid use disorders [8, 9], the study of dementia care in acute hospitals [10] and the establishment, implementation and evaluation of the matrices to achieve ready-everyday nursing standards [11]. However, it has rarely been used to analyze and manage the recall of medical devices.

The widespread application of medical devices and their complexity increase adverse events [12], so the analysis of recalls is particularly important. In 2008, Rich [13] listed 3 disturbing human factors that lead to medical device recalls, namely user expectations, device design and environment. And Gao et al. established a human–machine–environment interaction model to analyze the recalls of infusion pump [14]. Adverse events of medical device place a significant burden on the health of patients and the reputation of manufacturers [3, 15]. The analysis of recall events helps to control the risk of medical devices, protect the health of patients and provide some further insights into the better management of medical devices [14, 16].

To ensure the safety of surgery in bone and joint patients, we collected information on the recall numbers, recall classes, recall companies, recall classification and affected areas over the past decade. Based on the McKinsey 7S model and the adaptive model framework, we analyzed recall reasons of the two implants. On the results of this structured analysis, our study provides suggestions on how to avoid such implant recalls from the perspective of manufacturers, medical institutions and supervisions, as well as how to respond promptly to adverse events from the post-surveillance system perspective.

## Materials and methods

### Data collection

The medical device recall database is publicly reported on the website of the Chinese National Medical Products Administration (CNMPA), the US Food and Drug Administration (FDA), Healthy Canadians (HC) and the Australian Therapeutic Goods Administration (ATGA) [17–20]. We searched above databases for the recalls of osteosynthesis implant and Joint replacement implant between January 1, 2011, and June 30, 2021, and collected the information on the device name, recall time, class, manufacturer, device classification, affected areas and recall reasons. In the retrieval process, some recalls of the same devices are treated as one recall to avoid data duplication. Repeated recalls between countries were also excluded.

### Recall class

All recalls are classified into three categories based on the severity of the medical device defects. Class I recall is a medical device that could cause serious health injury or death. Class II recall refers to medical devices that are likely to cause temporary or reversible health harm or have the likelihood of causing serious health harm. Class III recall is a medical device that are unlikely to cause health harm but still require recall. Therefore, class I and class II recalls are combined into a category that may affect health, while class III recalls are considered as a normal recall.

### Factors causing the recall of medical devices

**Fig. 1:**
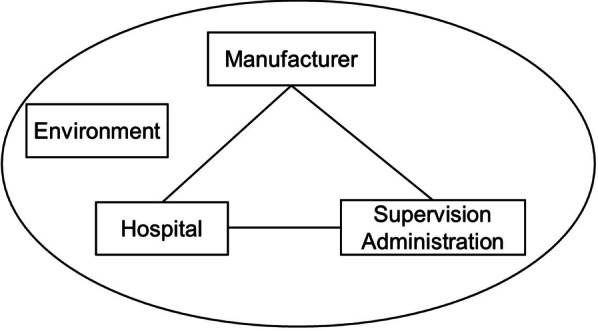
Main factors of medical device recall

The recall of medical device is caused by four factors (Fig. 1), including manufacturer, hospital, supervision administration and the environment [1, 9, 12–14, 21]. The manufacturer is responsible for the design and manufacture of the medical devices. Any problems during the pre-marketing sale can lead to a recall, such as mislabeled or wrong packaging. Hospital is the main place for utilizing the medical devices and is more likely to have the adverse events of the devices. Supervision administration must focus on all aspects of the design, manufacture and application in order to effectively avoid some problems. These three factors, from the manufacture to the application of medical devices, are correlated and interactive, and any negligence in any part may result in a recall. Moreover, environment and social factors may also affect the implementation of the above three factors.

**Fig. 2:**
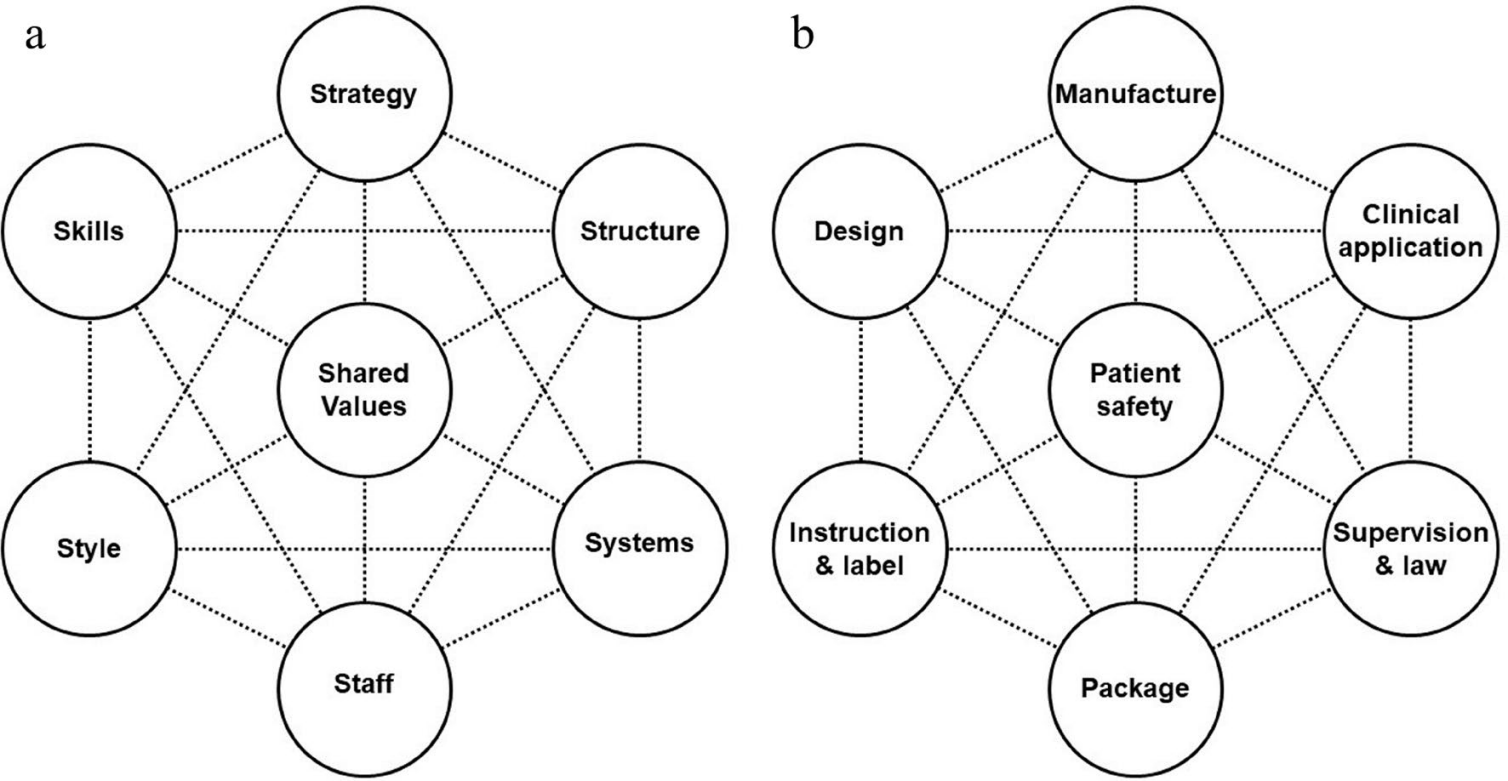
The McKinsey 7S model (**a**) and the new designed medical device risk management model (**b**)

Based on the McKinsey 7S model (Fig. 2A), we designed a new model to better manage the two implants. These seven factors were patient safety, design, manufacture, instructions and labels, packaging, clinical application and supervision and law (Fig. 2B). Except for patient safety, the manufacturer was responsible for the design, manufacture, instructions and labels and packaging, while clinical application and supervision and law were responsible by hospitals and supervision administrations, respectively.

**Fig. 3:**
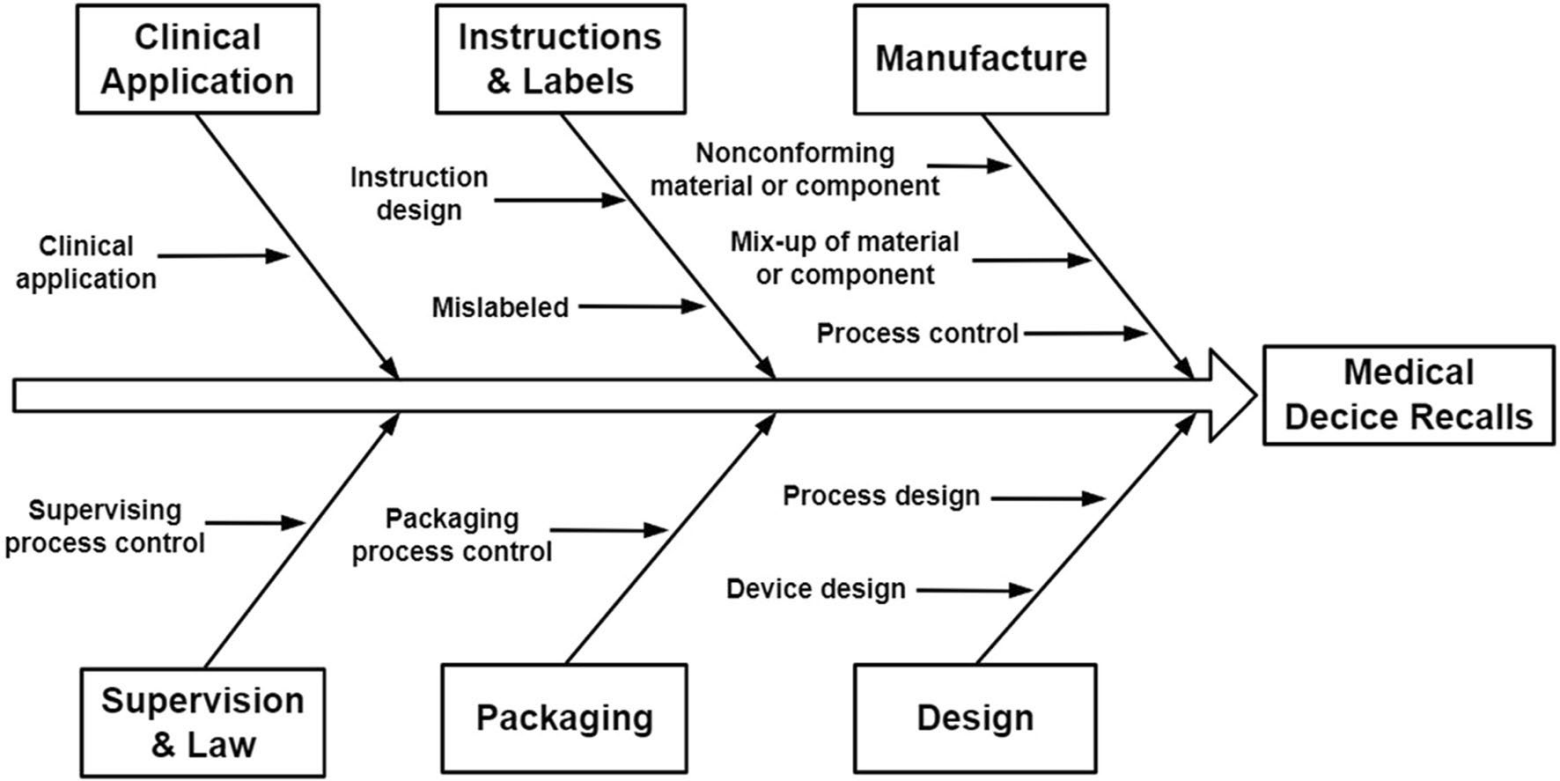
The fishbone diagram for the recall

To clarify the reasons more clearly, we further produced the fishbone diagram for the device recall (Fig. 3). The fish head represents the recall of the two implants; large fish bone represents the six factors except patient safety in the medical device risk management model; and each middle bone explains these six factors. Finally, the reasons are summarized and divided into 11 categories, including clinical application, supervising process control, device design, instruction design, mislabeled, mixed-up of material or component, nonconforming material or component, packaging process control, process control, process design and others. The definition of the reasons for each recall is given in Table 1.

**Table 1:** The definitions of the reasons for each recall

Reasons	Definitions
Clinical application	Due to some defects in the product 1. The operation is delayed or failed 2. The rate of recurrence or reoperation is increased
Supervising process control	The problems are found during the supervisory process
Device design	Due to the product design error 1. There are difficulties in the later production and manufacturing process (The Technology does not meet the requirements of the product size) 2. The product does not achieve the expected therapeutic effect in clinical application
Instruction design	Including 1. The contents of the instruction are wrong 2. The instruction does not match the products 3. The instruction is updated
Mislabeled	Including 1. The content on the label is wrong 2. The label does not match the product 3. The label is missing
Mix-up of material or component	The mix-up of different types of materials or components exists
Nonconforming material or component	Unqualified materials or components are used in the production process, including impure materials and errors in size, thickness, roughness of components, etc.
Packaging process control	Due to the packaging process error 1. There is packaging error, including omission or mix-up 2. The product packaging bag is poorly sealed 3. The sterile barrier is destroyed
Process control	Errors in production process result in unqualified and missing components or assembly errors
Process design	1. Production process design is mistaken 2. Some production or verification processes are missing
Others	Other reasons for not being classified

### Data analysis

Statistical analyses were performed using the Statistic Package for Social Sciences (SPSS, version 26.0, Chicago, IL) software. R × C tables were established for data difference comparison. Pearson chi-square test was used for analysis when the resulting categorical variables were disordered. If the actual observation frequency was less than 1, adjacent columns were merged. Rank-sum test was used when the resulting categorical variables were ordered hierarchies. A *p* value < 0.05 was considered significant for 2-tailed probability. Pairwise comparisons between multiple experimental groups were analyzed, and the hypothesis test level α was adjusted to *α*′ = *α*/number of comparisons.

## Results

### Time distribution of implant recall number

**Fig. 4:**
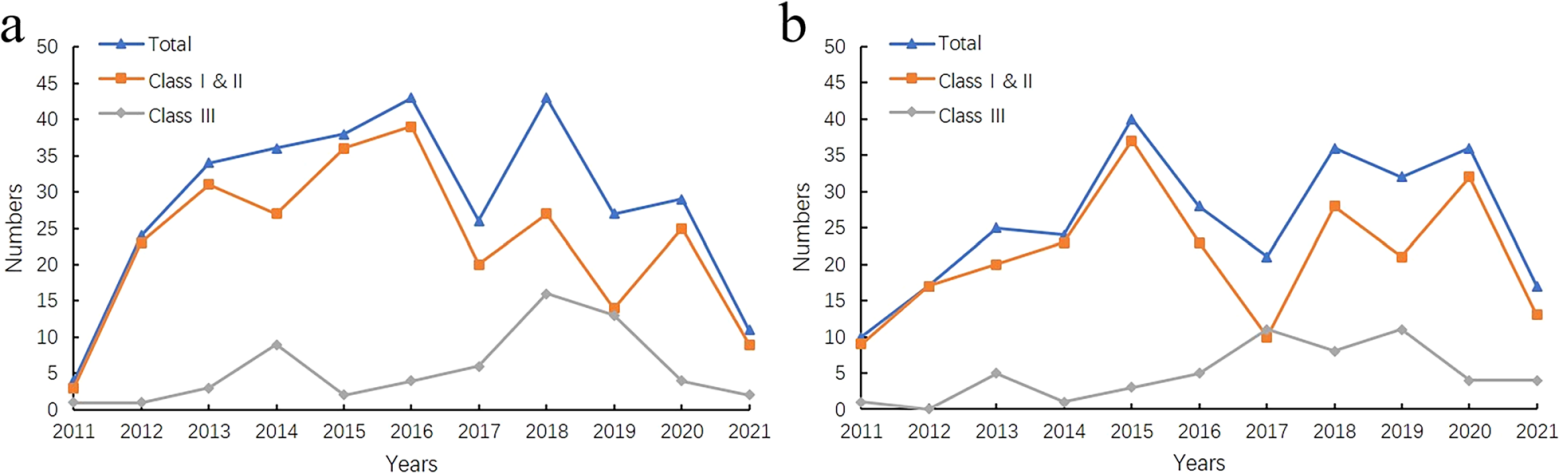
Time distribution of osteosynthesis implant recall number (**a**) and joint replacement implant recall number (**b**)

From January 1, 2011, to June 30, 2021, we collected 315 cases of osteosynthesis implant recalls (93 cases in China, 77 in the USA, 67 in Canada and 78 in Australia) and 286 cases of joint replacement implant recalls (104 cases in China, 68 in the USA, 50 in Canada and 64 in Australia). This decade was artificially divided into two periods, from 2011 to 2015 and 2016 to 2021. The recall number of the two implants increased in the first period and fluctuated significantly in the second period (Fig. 4). However, by comparing the total number of Class I and II recalls and Class III recalls, we found that the recalls in the second period were more than that in the first period for both osteosynthesis implant [(*c*^2^ = 8.854, *p* = 0.012), (Additional file 1: Table S1)] and joint replacement implant [(*c*^2^ = 12.697, *p* = 0.002), (Additional file 1: Table S2)]. The curve of class I and II recall number was roughly the same as the total recall curve, while the class III recall curve fluctuated at a low level.

### The distribution of implant recall class

The distribution of osteosynthesis implant recall class is almost the same as that of joint replacement implant (Table 2). The majority recall class of osteosynthesis implant was Class II accounted for 76.91%, 19.37% for Class III and 4.4% for Class I. Similarly, Class II of joint replacement implant recall was the most, accounted for 78.32%, followed by Class III for 18.53% and Class I for only 3.15%, without statistical differences between them (*Z* = − 0.072, *p* = 0.942). Pairwise comparisons between multiple experimental groups revealed no statistically significant differences between Class I versus Class II (*Z* = − 0.856, *p* = 0.392), Class I versus Class III (*Z* =  − 0.645, *p* = 0.519) and Class II versus Class III (*Z* = − 0.341, *p* = 0.733).

**Table 2:** Distribution of implant recall class

Recall class	Osteosynthesis implant		Joint replacement implant		*Z*	*p*
	Number	Percentage (%)	Number	Percentage (%)		
Class I	14	4.44	9	3.15	− 0.072	0.942
Class II	240	76.19	224	78.32		
Class III	61	19.37	53	18.53		

### The companies of implant recall

A total of 58 companies have recalled osteosynthesis implants, mostly published by five companies or manufacturers, including Zimmer Inc., Synthes Inc., Stryker Inc., Smith & Nephew Inc., Johnson & Johnson Inc., accounting for 62.86% (Additional file 1: Table S3). The recall of joint replacement implant was carried out by 35 companies, led by Zimmer Inc., Stryker Inc., Smith & Nephew Inc., Depuy Orthopedics Inc., Biomet Inc., accounting for 71.68% (Additional file 1: Table S4).

### The classification of implant recall

The most common product classification of osteosynthesis implant recall is single or multi-component metal bone fixation appliances and accessories, accounting for 63.49% (Additional file 1: Table S5). In the product categories of joint replacement implants, the hip prosthesis was ranked first, accounting for 50.00%, followed by the knee prosthesis, accounting for 36.36% (Additional file 1: Table S6).

### The affected areas of implant recall

**Fig. 5:**
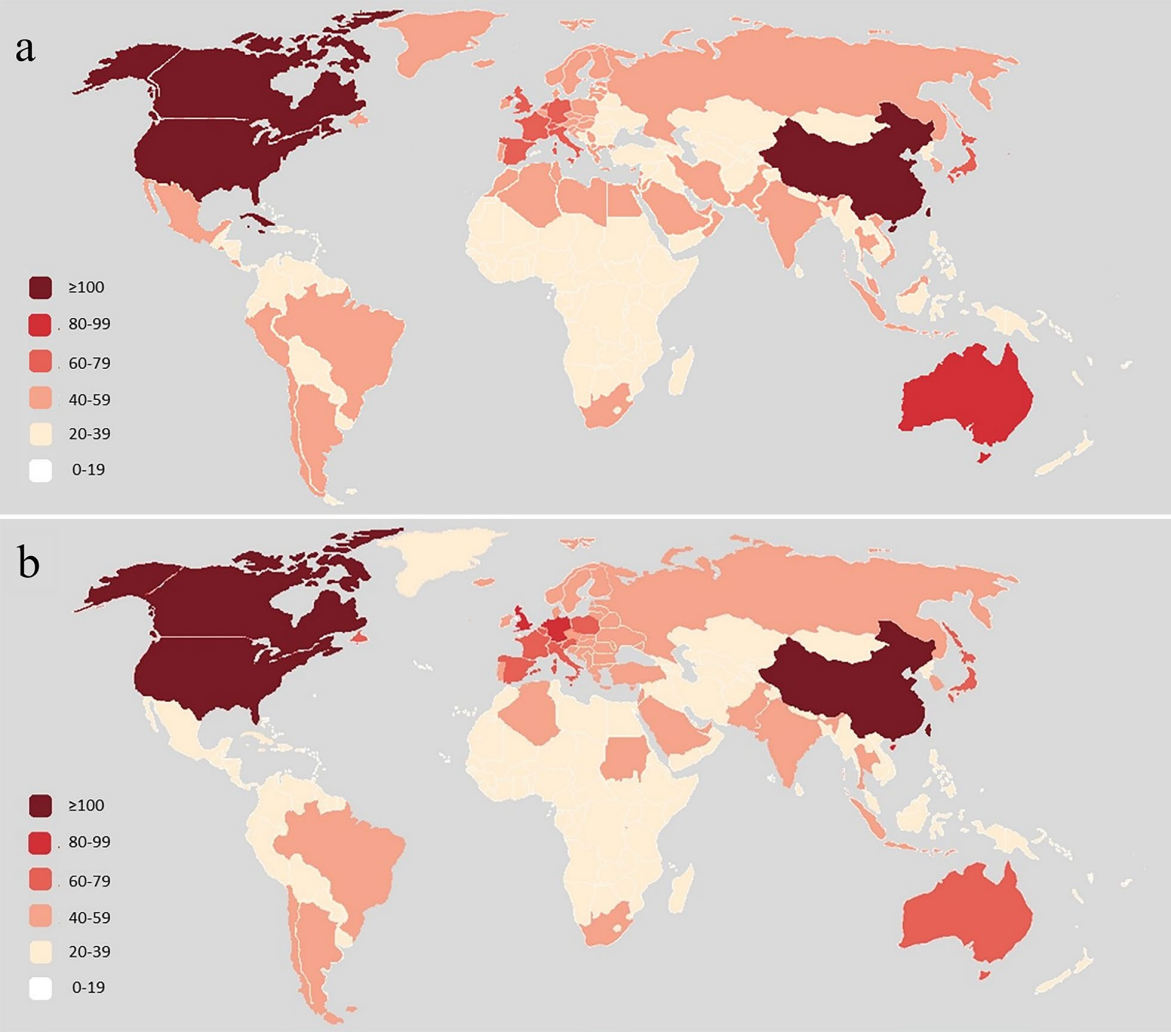
The affected areas of osteosynthesis implants (**a**) and joint replacement implants (**b**)

The recalls of the two implants have a broad impact, covering almost all continents (Fig. 5). Adverse events occur more frequently in Europe, North America, Australia and Southeast Asia, but less frequently in Africa, Latin America and West Asia.

### The reasons of implant recall

**Fig. 6:**
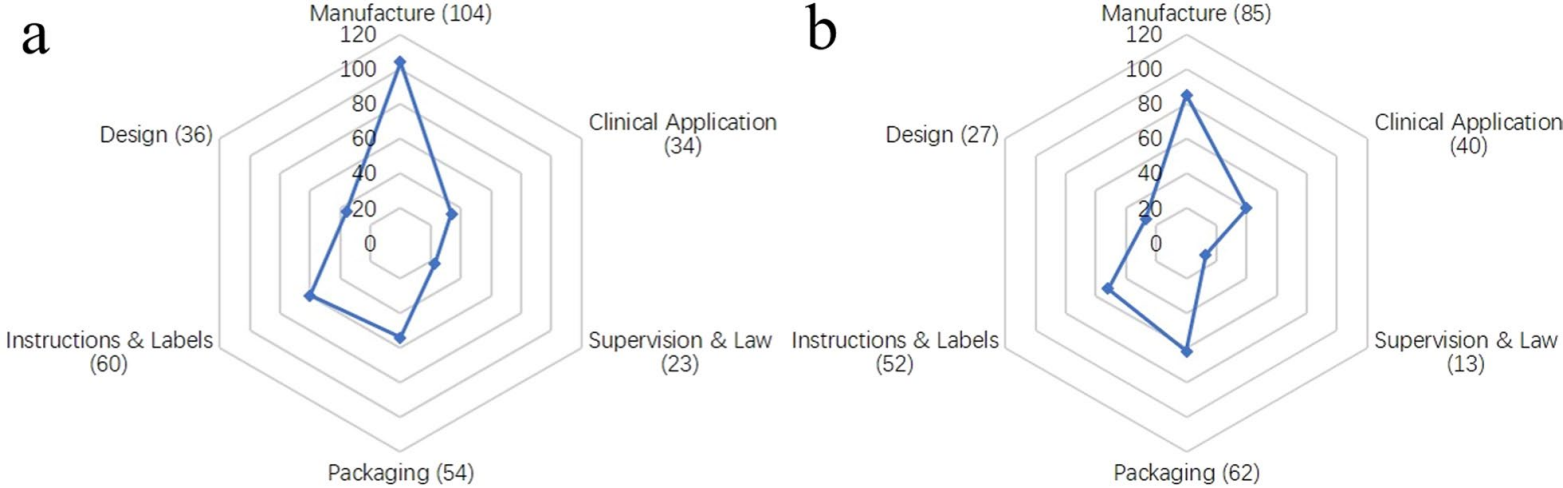
Main reasons for the recall of osteosynthesis implants (**a**) and joint replacement implants (**b**) based on risk management model

There are many reasons for recalls, including process control, packaging process control, mislabeled, nonconforming material or component, clinical application, device design, supervising process control, instruction design, mix-up of material or component, process design and others (Table 3). There was no statistically significant difference in the number of recalls between osteosynthesis implant and joint replacement implant for these reasons [(*c*^2^ = 8.052, *p* = 0.624), (Additional file 1: Table S7), (Fig. 6)]. The main causes for their co-existence are process control, packaging process control, mislabeled, nonconforming materials or components, clinical application and device design. (The number of recalls is greater than 25.) In addition, supervising process control and instruction design cannot be neglected. (The number of recalls is greater than 5.)

**Table 3:** Reasons of osteosynthesis implant and joint replacement implant recalls in China, America, Canada and Australia

Recalls			Process control	Packaging process control	Mislabeled	Nonconforming material or component	Clinical application	Device design	Supervising process control	Instruction design	Mix-up of material or component	Process design	Others
Osteosynthesis implant	Total	NO	56	54	52	44	34	33	23	8	4	3	4
		P (%)	17.78	17.14	16.51	13.97	10.79	10.48	7.30	2.54	1.27	0.95	1.27
	China	NO	20	14	22	20	4	0	6	2	2	1	2
		P (%)	21.51	15.05	23.66	21.51	4.30	0.00	6.45	2.15	2.15	1.08	2.15
	America	NO	10	2	11	9	6	17	7	1	1	2	1
		P (%)	14.93	2.99	16.42	13.43	8.96	25.37	10.45	1.49	1.49	2.99	1.49
	Canada	NO	12	12	5	9	11	9	7	1	1	0	0
		P (%)	17.91	17.91	7.46	13.43	16.42	13.43	10.45	1.49	1.49	0.00	0.00
	Australia	NO	14	17	14	6	13	7	3	4	0	0	0
		P (%)	17.95	21.79	17.95	7.69	16.67	8.97	3.85	5.13	0.00	0.00	0.00
Joint replacement implant	Total	NO	42	62	47	38	40	25	13	5	5	2	7
		P (%)	14.69	21.68%	16.43	13.29	13.99	8.74	4.55	1.75	1.75	0.70	2.45
	China	NO	15	27	28	10	3	7	4	2	3	1	4
		P (%)	14.42	25.96	26.92	9.62	2.88	6.73	3.85	1.92	2.88	0.96	3.85
	America	NO	10	17	8	9	9	9	6	0	0	0	0
		P (%)	14.71	25.00	11.76	13.24	13.24	13.24	8.82	0.00	0.00	0.00	0.00
	Canada	NO	10	12	4	8	7	3	2	1	2	0	1
		P (%)	20.00	24.00	8.00	16.00	14.00	6.00	4.00	2.00	4.00	0.00	2.00
	Australia	NO	7	6	7	11	21	6	1	2	0	1	2
		P (%)	10.94	9.38	10.94	17.19	32.81	9.38	1.56	3.13	0.00	1.56	3.13

NO., number; P (%), percentage

However, there were statistically significant differences in the number of osteosynthesis implant recalls due to these reasons in China, the USA, Canada and Australia [(c^2^ = 52.596, *p* = 0.000), (Additional file 1: Table S8)], and the number of joint replacement implant recalls in these four countries was also statistically different [(c^2^ = 49.629, *p* = 0.000), (Additional file 1: Table S9)]. Further analysis of osteosynthesis implant recalls caused by these reasons revealed statistical differences in numbers between China and the USA (c^2^ = 28.226, *p* = 0.000), China and Canada (c^2^ = 24.029, *p* = 0.001) and the USA and Australia (c^2^ = 21.211, *p* = 0.002), while only China and Australia had a statistical difference in the number of joint replacement implant recalls (c^2^ = 38.347, *p* = 0.000).

## Discussion

The recalls number of osteosynthesis implants and joint replacement implants fluctuated from 2016 to 2021, and the overall number remained rising and significantly higher than that from 2011 to 2015. However, the increasing recall number does not mean that the safety and efficacy of medical devices are decreasing. Steven Kurtz et al. had predicted that demand for knee arthroplasty in the USA was expected to increase by 673% between 2005 and 2030 [22]. The value of the entire Asian medical device market is expected to reach 3.719 billion dollars by 2028 [23]. The longer the implant stays in the body, the more metal ions are released, affecting human health and leading to higher revision rates [24]. Therefore, the problems of devices implanted a decade ago may have gone undetected until now, which is why the number of recalls has increased from 2016 to 2021.

Further integration in the different branches of the same company reveals that recalls number of Zimmer Biomet, Johnson & Johnson, Smith & Nephew and Stryker accounted for more than 70% of the total. According to the 2018 data of the Orthopedic Big Data Editorial Office (OBDE), these four companies are all ranked in the first tier of the top 100 global companies, accounting for 58% of the industry’s $51.2 billion market share [3, 25]. In addition, recall awareness is another major factor. Developed countries and regions such as the USA, Canada and the European Union have established a complete medical device recall system earlier [12], which is beneficial for enterprises to establish quality system and actively promote the recall of defective products.

The recalls of the two implants have had a widespread impact around the world, covering almost all fields. Europe, the USA, Australia and Southeast Asia had higher incidences, while Africa and the Middle East had lower incidences. We found that most economically advanced countries have been affected by the recall, while the impact on less developed countries is smaller. This can be explained by the fact that developed countries can afford higher medical expenses and promote utilizing high-priced medical devices, such as implants [26]. In addition, the distribution of recall events is consistent with the distribution of bone disease prevalence. High-income countries such as Europe and the USA are the most affected by skeletal diseases with 441 million people, followed by the WHO Western Pacific region with 427 million and Southeast Asia with 369 million [27].

There was no statistical difference in the recall reasons between the two implants. The main common causes were process control, packaging process control and nonconforming materials or components. Most implants are highly precise and small with complex structures and multiple components, which are the objective factors prone to these problems [28]. Except for small probability events, nonconforming materials or components are mainly caused by subjective factors, including carelessness in the procurement and sorting process, improper packaging in process control, especially the destruction of sterile barriers [13]. In addition, the choice of surgical methods and doctors’ awareness of reporting are also important factors affecting the curative effects [21].

In contrast, the distribution of recall reasons varies among countries. The proportion of device design is significantly higher in the USA than in other three countries, which is consistent with the fact that most of the top giants are American companies. In China, labeling errors are very prominent. On the one hand, most of China’s mid-to-high-end medical devices are excessively dependent on imports [29]. On the other hand, the regulatory authorities’ understanding and implementation standards of Article 42 of the Regulations on the Supervision and Administration of Medical Devices are not consistent [30]. However, the laws and regulations have only prohibited provisions and no provisions for effective accountability, which makes them less deterrent.

The safety and efficacy of the medical device depend on whether the manufacturers, medical institutions and supervisions can assume their responsibilities. Our results showed that the connection between manufacturers is not ideal, especially in process control, packaging process control and labeling errors. Meanwhile, many medical workers are reluctant to actively report adverse events, believing it is not their responsibility [21]. Combining the medical device risk management model with our results, we emphasized eight recommendations to improve device safety. Suggestions 1–4 apply to manufacturers, 5–6 to medical institutions and 7–8 to supervisions.

1. Materials and Components. For biodegradable materials, special attention should be paid on whether the implants will release harmful products such as the wear and corrosion during the biodegradation process. The company should set up the corresponding inspection department, strictly screen components, timely eliminate unqualified components and prevent them from flowing into the production line. In addition, it is also a good choice to build a reasonable and convenient component classification and placement system, which can effectively avoid confusion of materials or components.
2. Labels and Instructions. Detailed and readable labels and instructions are important in the regulation of medical device. When writing labels and instructions, the producer should consider the later identification, use and supervision to ensure that labels and instructions are accurate and effective. At the same time, the manufacturers should actively respond to the regulatory requirements and improve the inspection system for mismatched labels and instructions.
3. Packaging Process Control. Manufactures must strictly sterilize packaging materials and packaging process to ensure that sterility barriers are not damaged. At the same time, qualified full-time inspectors are required to undertake environmental monitoring, biological inspection and other quality control work. During the packing process, the staff should ensure that labels and glue on the surface are completely isolated from the devices.
4. Data Integrity of Medical Devices. The manufactures should establish a record control system through electronic information technology, including the label, storage, retrieval, preservation period and application requirements of the devices. In the event of device failure, manufacturers can use information retrieval to track faulty devices and recall it if necessary.
5. Purchase and Use. Medical institutions should standardize the procurement process to avoid unqualified devices entering the hospital. Before purchasing, the use of the product in the market should be fully investigated. At the same time, the relevant superintendent should be trained to ensure the quantity and quality of devices handover. Operators should strictly follow the instructions when using the device. In addition, aseptic operation is also an important step.
6. Training of Medical Workers. Hospitals should pay attention to the professional skills training of operators to improve the success rate of surgery. And it is important to increase the responsibility of healthcare professionals and timely reporting of adverse events to regulators and manufacturers to reduce continue risks. In addition, strengthening postoperative follow-up, dynamically mastering the changes of medical devices and patients’ use feelings, is beneficial to ensure the long-term use of medical equipment and timely detection of possible adverse events [31–33].
7. Premarket Safety Assessment. A series of premarket safety assessments of medical devices are required, including the strength of connections, joints or sealing, tolerability and mechanical properties of implants. Besides, biocompatibility assessment and histopathological testing of the implants can not only detect toxic effects, but also assess the efficacy of the product. The assessment agency staff should report risk factors in a timely manner so that the producers can eliminate possible risks before the product is officially marketed.
8. Reporting and Regulating. Regulators should strengthen the supervision and develop a fast and simple medical device adverse event reporting system and fully ensure the transparency of recall database information. Opening a special reporting line or reporting mailbox to quickly grasp adverse events is conducive to improving the reporting rate of adverse events.

This study comprehensively analyzed the recalls of osteosynthesis implants and joint replacement implants in the aspect of time periods, recall classes, recall companies, recall classification, affected areas, main countries and recall reasons, which not only helps to improve the safety and efficacy of medical devices, but also provides some useful insights for the risk management of medical devices. In addition, we designed a new medical device risk management model based on the McKinsey 7S model and try to combine it with fishbone model to better analyze recall reasons of these two implants.

### Limitations

The main limitation is that many other types of recalls were not reported in our study. That is because these recalls are not reported to regulators, but directly to medical device providers. In addition, there are not sufficient data for further study. This is because the mandatory recall is not strong enough, some companies do not voluntarily recall defective devices, and consumers’ ignorance of the medical device recall system may also result in some defective devices not being reported.

## Conclusions

To avoid the recalls of osteosynthesis implants and joint replacement implants, manufacturers should strictly select implant materials and components, develop detailed labels and instructions, severely control the packaging process and establish the integrity of medical device data. Medical institutions should standardize procurement procedures, use qualified equipment and train medical workers in professional skills. It also requires supervisions to conduct premarket safety assessments of medical devices. In addition, regulators should strengthen supervision and establish reporting systems to deal with the occurrence of adverse events promptly.

## Supplementary Information

**Additional file 1. Table S1.** Time distribution of osteosynthesis implant recall number. **Table S2.** Time distribution of joint replacement implant recall number. **Table S3.** The companies of osteosynthesis implant recall. **Table S4.** The companies of joint replacement implant recall. **Table S5.** The classification of osteosynthesis implant recall. **Table S6.** The classification of joint replacement implant recall. **Table S7.** Reasons for the recall of osteosynthesis implant and joint replacement implant. **Table S8.** Reasons for the recall of osteosynthesis implant in China, USA, Canada and Australia. **Table S9.** Reasons for the recall of joint replacement implant in China, USA, Canada and Australia.

## Data Availability

All datasets analyzed during this study are available from the corresponding author upon reasonable request.

## Abbreviations

CNMPA: Chinese National Medical Products Administration
FDA: The US Food and Drug Administration
HC: Healthy Canadians
ATGA: The Australian Therapeutic Goods Administration
OBDE: The Orthopedic Big Data Editorial Office

## References

[1] Shukla S, Gupta M, Pandit S, Thomson M, Shivhare A, Kalaiselvan V (2020). Implementation of adverse event reporting for medical devices. India Bull World Health Organ.

[2] Akoh CC, Chen J, Kadakia R, Park YU, Kim H, Adams SB (2021). Adverse events involving hallux metatarsophalangeal joint implants: analysis of the United States Food and Drug Administration data from 2010 to 2018. Foot Ankle Surg Off J Eur Soc Foot Ankle Surg.

[3] Vajapey SP, Li M (2020). Medical device recalls in orthopedics: recent trends and areas for improvement. J Arthroplasty.

[4] Agarwal R, Garcia AJ (2015). Biomaterial strategies for engineering implants for enhanced osseointegration and bone repair. Adv Drug Deliv Rev.

[5] ISO 14602: 1998, Non-active surgical implants-Implants for osteosynthesis-particular requirements, IDT

[6] ISO 21534: 2002 , Non-active surgical implantJoint replacement implants-particular requirements , IDT

[7] Waterman RH, Peters TJ, Phillips JR (1980). Structure is not organization. Bus Horiz.

[8] Kawasaki S, Dunham E, Mills S, Kunkel E, Gonzalo JD (2021). The opioid epidemic: mobilizing an academic health center to improve outcomes. J Subst Abuse Treat.

[9] Sokol R, Schuman-Olivier Z, Batalden M, Sullivan L, Shaughnessy AF (2020). A change management case study for safe opioid prescribing and opioid use disorder treatment. J Am Board Fam Med.

[10] Scerri A, Innes A, Scerri C (2020). Dementia care in acute hospitals-A qualitative study on nurse managers' perceived challenges and solutions. J Nurs Manag.

[11] Ratsch A, Sewell F, Pennington A (2019). Developing and testing a matrix to achieve ready-everyday nursing standards (RENS): an observational study protocol. BMJ Open.

[12] Sarkissian A (2018). An exploratory analysis of US FDA Class I medical device recalls: 2014–2018. J Med Eng Technol.

[13] Rich S (2008). How human factors lead to medical device adverse events. Nursing.

[14] Gao X, Wen Q, Duan X, Jin W, Tang X, Zhong L (2019). A Hazard analysis of class I recalls of infusion pumps. JMIR Hum Factors.

[15] Elmallah RK, Cherian JJ, Meneghini RM, Hozack WJ, Westrich GH, Mont MA (2016). How to approach a recalled dual modular hip implant: an update. J Arthroplasty.

[16] Mahmoud K, Metikala S, O'Connor KM, Farber DC (2021). Adverse events related to total ankle replacement devices: an analysis of reports to the United States Food and Drug Administration. Int Orthop.

[17] Administration. CNMP. Medical devices recalls. https://www.nmpa.gov.cn/xxgk/chpzhh/ylqxzhh/index.html Assessed 2 July 2021. 2021.

[18] FDA. Medical device recalls. https://www.accessdata.fda.gov/scripts/cdrh/cfdocs/cfres/res.cfm Assessed 2 July 2021. 2021.

[19] Canadians. H. Recalls and safety alerts. https://recalls-rappels.canada.ca/en/search/site?f%5B0%5D=recall_type%3A255 Assessed 2 July 2021. 2021.

[20] Administration. ATG. System for Australian recall actions. https://apps.tga.gov.au/PROD/SARA/arn-entry.aspx Assessed 2 July 2021. 2021.

[21] Gagliardi AR, Ducey A, Lehoux P, Turgeon T, Ross S, Trbovich P (2018). Factors influencing the reporting of adverse medical device events: qualitative interviews with physicians about higher risk implantable devices. BMJ Qual Saf.

[22] Kurtz S, Ong K, Lau E, Mowat F, Halpern M (2007). Projections of primary and revision hip and knee arthroplasty in the United States from 2005 to 2030. J Bone Joint Surg Am.

[23] Jakovljevic M, Wu W, Merrick J, Cerda A, Varjacic M, Sugahara T (2021). Asian innovation in pharmaceutical and medical device industry—beyond tomorrow. J Med Econ.

[24] Maurer-Ertl W, Friesenbichler J, Holzer LA, Leitner L, Ogris K, Maier M (2017). Recall of the ASR XL head and hip resurfacing systems. Orthopedics.

[25] 12 Chinese enterprises were shortlisted as the top 100 global orthopedic medical device companies. Brand Stand 2019;5:42–47.

[26] Mbachu C, Okoli C, Onwujekwe O, Enabulele F (2018). Willingness to pay for antiretroviral drugs among HIV and AIDS clients in south-east Nigeria. Health Expect.

[27] Cieza A, Causey K, Kamenov K, Hanson SW, Chatterji S, Vos T (2020). Global estimates of the need for rehabilitation based on the Global Burden of Disease study 2019: a systematic analysis for the Global Burden of Disease Study 2019. The Lancet.

[28] Yang Y, He C, Dianyu E, Yang W, Qi F, Xie D (2020). Mg bone implant: features, developments and perspectives. Mater Des.

[29] Jun L (2015). Analysis on the reasons why imported medical devices monopolize China's medium and high-end market. Drug Dev Manag.

[30] Feng Z (2018). The quality of the label of imported medical equipment bears the brunt. Med Econ.

[31] Benelli G, Maritato M, Cerulli Mariani P, Sasso F (2019). Revision of ASR hip arthroplasty: analysis of two hundred and ninety six recalled patients at seven years. Int Orthop.

[32] Galea VP, Rojanasopondist P, Matuszak SJ, Connelly JW, Ray GS, Madanat R (2021). Current evidence from a worldwide, multicentre, follow-up study of the recalled Articular Surface Replacement Hip System. Hip Int.

[33] Nantel J, Termoz N, Centomo H, Lavigne M, Vendittoli PA, Prince F (2008). Postural balance during quiet standing in patients with total hip arthroplasty and surface replacement arthroplasty. Clin Biomech (Bristol, Avon).

