# Supplementary material for: Investigation and analysis of four countries’ recalls of osteosynthesis implants and joint replacement implants from 2011 to 2021
Source: J Orthop Surg Res. 2022 Oct 7;17:443. doi: 10.1186/s13018-022-03332-w (PMC9547470; doi:10.1186/s13018-022-03332-w)
Supplement: Supplementary file 1 — Additional file 1. Table S1. Time distribution of osteosynthesis implant recall number. Table S2. Time distribution of joint replacement implant recall number. Table S3. The companies of osteosynthesis implant recall. Table S4. The companies of joint replacement implant recall. Table S5. The classification of osteosynthesis implant recall. Table S6. The classification of joint replacement implant recall. Table S7. Reasons for the recall of osteosynthesis implant and joint replacement implant. Table S8. Reasons for the recall of osteosynthesis implant in China, USA, Canada and Australia. Table S9. Reasons for the recall of joint replacement implant in China, USA, Canada and Australia. [file 13018_2022_3332_MOESM1_ESM.docx]

**Supplementary Table 1** Time distribution of osteosynthesis implant recall number

| Recall year | total | Class I & II | Class III | χ^2^ | *p* |
| --- | --- | --- | --- | --- | --- |
| 2011-2015 (NO.) | 136 | 120 | 16 | 8.854 | 0.012 |
| 2016-2021 (NO.) | 179 | 134 | 45 |  |  |

NO. = number

**Supplementary Table 2** Time distribution of joint replacement implant recall number

| Recall year | total | Class I & II | Class III | χ^2^ | *p* |
| --- | --- | --- | --- | --- | --- |
| 2011-2015 (NO.) | 116 | 106 | 10 | 12.697 | 0.002 |
| 2016-2021 (NO.) | 170 | 127 | 43 |  |  |

NO. = number

**Supplementary Table** **3** The companies of osteosynthesis implant recall

| Recall Company | Number |
| --- | --- |
| Zimmer, Inc. | 70 |
| Synthes, Inc. | 55 |
| Smith & Nephew, Inc. | 26 |
| Stryker, Inc. | 26 |
| Johnson & Johnson Medical Pty Ltd t/a Depuy Synthes | 21 |
| Biomet, Inc. | 17 |
| DePuy Orthopedics Inc | 11 |
| Exactech, Inc. | 5 |
| Pega Medical Inc. | 5 |
| Unrecorded | 5 |
| OrthoPediatrics Corp | 4 |
| Tornier Pty Ltd | 4 |
| Arthrex, Inc. | 3 |
| Emergo Asia Pacific Pty Ltd T/a Emergo Australia | 3 |
| Medacta Pty Ltd | 3 |
| Medtronic Inc | 3 |
| NuVasive, Inc | 3 |
| PioneerSurgical Technology, Inc. | 3 |
| Acumed LLC | 2 |
| Integra LifeSciences Corporation | 2 |
| Jiangsu IDEAL MEDICAL Science and Technology Co., Ltd | 2 |
| Lima Orthpedics Technology Pty Ltd | 2 |
| Orthofix, Inc | 2 |
| Shanghai Pudong Jinhuan Medical Products Co., Ltd | 2 |
| Signature Orthopedics Pty Ltd | 2 |
| Wright Medical Australia Pty Ltd | 2 |
| aap Implantate AG | 1 |
| Advanced Surgical Design & Manufacture Ltd | 1 |
| Aesculap AG | 1 |
| Akva Surgical | 1 |
| Anjon Holdings | 1 |
| Aplus（Shanghai）trading Co., Ltd | 1 |
| Baxter Healthcare Pty Ltd | 1 |
| Bioplate, Inc. | 1 |
| CareFusion Australia 316 Pty Ltd | 1 |
| Ceraver Les Laboratoires Osteal médical | 1 |
| Evolution Surgical Pty Ltd | 1 |
| EXP Pharmaceutical Services Corp | 1 |
| General Care（Shanghai） Co., Ltd | 1 |
| Howmedica Osteonics Corporation | 1 |
| Implant Innovations Inc. | 1 |
| In2Bones, SAS | 1 |
| Integra Neurosciences Pty Ltd | 1 |
| Kalaparna Pty Ltd | 1 |
| LIBEIER An AK MEDICAL Company | 1 |
| Life Healthcare Pty Ltd | 1 |
| Medos International SARL | 1 |
| Merete Medical GmbH | 1 |
| Newdeal Sas Imeuble | 1 |
| Nextremity Solutions | 1 |
| Orthovita Inc. | 1 |
| Scientx Australia | 1 |
| Shanghai MiroPort Orthopedics Co., Ltd | 1 |
| Shanghai Puwei Medical Instrument Factory Co., Ltd | 1 |
| Shitaibao Orthopedic Implant Co., Ltd | 1 |
| Steris Corporation | 1 |
| TriMed Inc. | 1 |
| Vascutek Limited a Terumo Compony | 1 |

**Supplementary Table 4** The companies of joint replacement implant recall

| Recall Company | Number |
| --- | --- |
| Zimmer, Inc. | 88 |
| Smith & Nephew, Inc. | 53 |
| Biomet, Inc. | 32 |
| Depuy Orthopedics Inc | 17 |
| Stryker, Inc | 15 |
| Howmedica Osteonics Corp. | 13 |
| MicroPort Orthopedics, Inc. | 7 |
| Global Orthopedic Technology Pty Ltd | 5 |
| Medacta, Inc | 5 |
| Wright Medical Technology Inc | 5 |
| Amplitude Australia Pty Ltd | 4 |
| Corin Ltd | 4 |
| Encore Medical, Lp | 4 |
| Tornier, Inc | 4 |
| Bangmei（Shanghai）Co., Ltd | 3 |
| Exactech, Inc. | 3 |
| Johnson & Johnson Medical Pty Ltd t/a Depuy Synthes | 3 |
| Ceraver Les Laboratoires Osteal medical | 2 |
| Tian Xin Fu（Beijing）Medical Appliance Co., Ltd | 2 |
| Unrecorded | 2 |
| Advanced Surgical Design & Manufacture Ltd | 1 |
| Arthrosurface, Inc. | 1 |
| B Braun Australia Pty Ltd | 1 |
| Integra LifeSciences Corp. | 1 |
| Integra Neurosciences Pty Ltd | 1 |
| Lima Orthpedics Technology Pty Ltd | 1 |
| Mathys Orthopedics Pty Ltd | 1 |
| MAXONIQ Pty Ltd | 1 |
| OMNI life science Inc. | 1 |
| Orthotech Pty Ltd | 1 |
| RQ Solutions Medical Devices Distribution Support Pty Ltd | 1 |
| Signature Orthopedics Pty Ltd | 1 |
| Surgical Specialties Pty Ltd | 1 |
| United Medical Device Co., Ltd | 1 |
| France serf | 1 |

**Supplementary Table 5** The classification of osteosynthesis implant recall

| Product classification | Number |
| --- | --- |
| Single/multiple component metallic bone fixation appliances and accessories | 200 |
| Metal intramedullary device | 80 |
| Metal bone fasteners with smooth surfaces or threads | 31 |
| Metal fixing Cerclage device | 4 |

**Supplementary Table 6** The classification of joint replacement implant recall

| Product classification | Number |
| --- | --- |
| hip prosthesis | 143 |
| Knee prosthesis | 104 |
| Shoulder prosthesis | 23 |
| Elbow prosthesis | 8 |
| Ankle prosthesis | 6 |
| Temporomandibular joint prosthesis | 2 |

**Supplementary Table 7** Reasons for the recall of osteosynthesis implant and joint replacement implant

| Implants | Process control | Packaging process control | Mislabeled | Nonconforming material or component | Clinical application | Device design | Supervising process control | Instruction design | Mix-up of material or component | Process design | Others | χ^2^ | *p* |
| --- | --- | --- | --- | --- | --- | --- | --- | --- | --- | --- | --- | --- | --- |
| Osteosynthesis implant (NO.) | 54 | 52 | 56 | 34 | 44 | 33 | 23 | 8 | 4 | 3 | 4 | 8.052 | 0.624 |
| Joint replacement implant (NO.) | 62 | 47 | 42 | 40 | 38 | 25 | 13 | 5 | 5 | 2 | 7 |  |  |

NO. = number

**Supplementary Table 8** Reasons for the recall of osteosynthesis implant in China, USA, Canada and Australia

| Countries | Process control | Packaging process control | Mislabeled | Nonconforming material or component | Clinical application | Device design | Supervising process control | Instruction design | Mix-up of material or component | Process design | Others | χ^2^ | *p* |
| --- | --- | --- | --- | --- | --- | --- | --- | --- | --- | --- | --- | --- | --- |
| China (NO.) | 14 | 22 | 20 | 4 | 20 | 0 | 6 | 2 | 2 | 1 | 2 | 52.596 | 0.000 |
| USA (NO.) | 2 | 11 | 10 | 6 | 9 | 17 | 7 | 1 | 1 | 2 | 1 |  |  |
| Canada (NO.) | 12 | 5 | 12 | 11 | 9 | 9 | 7 | 1 | 1 | 0 | 0 |  |  |
| Australia (NO.) | 17 | 14 | 14 | 13 | 6 | 7 | 3 | 4 | 0 | 0 | 0 |  |  |

NO. = number

Note - Since the actual observation frequency is less than 1, the device design is merged with adjacent supervising process control, and the instruction design is merged with adjacent mix-up of material or component, process design and others.

**Supplementary Table 9** Reasons for the recall of joint replacement implant in China, USA, Canada and Australia

| Countries | Process control | Packaging process control | Mislabeled | Nonconforming material or component | Clinical application | Device design | Supervising process control | Instruction design | Mix-up of material or component | Process design | Others | χ^2^ | *p* |
| --- | --- | --- | --- | --- | --- | --- | --- | --- | --- | --- | --- | --- | --- |
| China (NO.) | 27 | 28 | 15 | 3 | 10 | 7 | 4 | 2 | 3 | 1 | 4 | 49.629 | 0.000 |
| USA (NO.) | 17 | 8 | 10 | 9 | 9 | 9 | 6 | 0 | 0 | 0 | 0 |  |  |
| Canada (NO.) | 12 | 4 | 10 | 7 | 8 | 3 | 2 | 1 | 2 | 0 | 1 |  |  |
| Australia (NO.) | 6 | 7 | 7 | 21 | 11 | 6 | 1 | 2 | 0 | 1 | 2 |  |  |

NO. = number

Note - Since the actual observation frequency is less than 1, the supervising process control is merged with adjacent instruction design, mix-up of material or component, process design and others.
